# The (non-)replicability of regulatory resource depletion: A field report employing non-invasive brain stimulation

**DOI:** 10.1371/journal.pone.0174331

**Published:** 2017-03-31

**Authors:** Franziska Emmerling, Carolien Martijn, Hugo J. E. M. Alberts, Alix C. Thomson, Bastian David, Daniel Kessler, Teresa Schuhmann, Alexander T. Sack

**Affiliations:** 1 Department of Cognitive Neuroscience, Maastricht University, Maastricht, The Netherlands; 2 Maastricht Brain Imaging Center, Maastricht, The Netherlands; 3 Department of Experimental Psychology, Oxford University, Oxford, United Kingdom; 4 Department of Clinical Psychological Science, Maastricht University, Maastricht, The Netherlands; 5 Center for Economics and Neuroscience, University of Bonn, Bonn, Germany; 6 Department of Psychiatry, University of Michigan, Ann Arbor, MI, United States of America; University Medical Center Goettingen, GERMANY

## Abstract

Cognitive effort and self-control are exhausting. Although evidence is ambiguous, behavioural studies have repeatedly suggested that control-demanding tasks seem to deplete a limited cache of self-regulatory resources leading to performance degradations and fatigue. While resource depletion has indirectly been associated with a decline in right prefrontal cortex capacity, its precise neural underpinnings have not yet been revealed. This study consisted of two independent experiments, which set out to investigate the causal role of the right dorsolateral prefrontal cortex (DLPFC) in a classic dual phase depletion paradigm employing non-invasive brain stimulation. In Experiment 1 we demonstrated a general depletion effect, which was significantly eliminated by anodal transcranial Direct Current Stimulation to the right DLPFC. In Experiment 2, however, we failed to replicate the basic psychological depletion effect within a second independent sample. The dissimilar results are discussed in the context of the current ‘replication crisis’ and suggestions for future studies are offered. While our current results do not allow us to firmly argue for or against the existence of resource depletion, we outline why it is crucial to further clarify which specific external and internal circumstances lead to limited replicability of the described effect. We showcase and discuss the current inter-lab replication problem based on two independent samples tested within one research group (intra-lab).

## Introduction

Humans are capable of self-regulation or self-control. Good self-regulatory control is crucial for economic and interpersonal success [1–4] and its failure can entail negative consequences such as over-eating, substance abuse, violent behaviour, or procrastination [5–7]. According to the strength model [8], which has seen controversy and debate in recent literature [9], self-control draws on a fixed and limited resource, akin to strength or energy. After an act of self-regulation, the resource becomes temporarily depleted leaving the individual in a state of *ego depletion*. Consequently, performance on a subsequent self-control act will be impaired due to a lack of resources. Empirical evidence for the ego depletion effect is ambiguous. Although meta-analyses showed that over 100 studies have replicated the ego depletion effect in different domains [1], some authors have suggested that the evidence on the effect was over-estimated due to the publication-bias [10]. More recent massive systematic empirical efforts have even failed to replicate the effect [2].

The exhaustion of cognitive capacity is metaphorically paralleled with the exhaustion of physical strength [6]. While the physiological mechanisms underlying the restriction of muscle energy are well understood, there is no comparably understood mechanism in the brain. Such a mechanism or ‘self-control battery’ would require a neurobiological substrate within an identifiable neural system and should be accessible for neuroscientific investigation and directly brain-based experimental manipulation.

Empirical investigations addressing the neural correlates of regulatory resource depletion are scarce. In an attempt to unveil the neural correlates of self-control, Heatherton and Wagner [11] proposed a model that differentiates between two basic entities within the brain: a subcortical impulse system and a prefrontal control system. The former system, comprising of mainly subcortical structures (i.e. amygdala and nucleus accumbens), reacts immediately to basic drives and urges. This instant reaction is regulated by a control system within dorsolateral prefrontal and anterior cingulate cortexes. Any imbalance in favour of the subcortical system within this subcortical-prefrontal circuit leads to self-regulation failure. Such imbalance can either arise through an overstimulation of the subcortical system due to overwhelming external triggers or through a breakdown of the prefrontal control system due to overstrain. Specifically, the latter process could be a neural mechanism underlying regulatory resource depletion.

Support for the claim that resource depletion may arise from overstraining the prefrontal cortex was offered by Sripada and colleagues [12]. In their study, the ego depletion effect decreased after administration of a catecholamine-boosting agent–i.e., Methylphenidate. This drug is known to affect prefrontal brain circuits [13]. The intervention likely supported the prefrontal control system, ameliorating overstrain, which resulted in decreased resource depletion. Imaging studies have supported this interpretation and have revealed an association between regulatory resource depletion and reduced activity in dorsolateral and ventromedial prefrontal cortex, as well as reduced connectivity between these regions and subcortical structures [14,15].

To date, the few studies that have addressed the neural link between self-regulation failure and regulatory resource depletion relied on psychopharmacological interventions and functional imaging. So far, however, the causal role of the prefrontal cortex in self-regulation failure and regulatory resource depletion has not been investigated by manipulating its function directly. The primary aim of the present experiments was to fill this gap by elucidating the neural correlates of self-regulation failure by attempting to selectively reduce depletion using transcranial Direct Current Stimulation (tDCS). TDCS is believed to manipulate the excitability of cortical brain regions by altering membrane potentials [16,17]. It is a promising tool to investigate the role of certain brain areas in a given cognitive process and to subsequently develop directly brain-based interventions. In Experiment 1, participants were randomised into two groups; the first group received stimulation anodal stimulation of the right dorsolateral prefrontal cortex, whereas the second group received placebo stimulation. Participants were subjected to two sessions consisting of two phases each (classic dual phase paradigm), in which the self-regulatory demands of the phase I task were varied to manipulate the level of induced depletion. In phase II, performance on a task demanding high regulatory control served as the primary outcome. For the placebo stimulation condition, we hypothesised that participants would show reduced task performance in phase II after induction of high depletion in phase I due to temporarily depleted resources (i.e., a depletion effect). Conversely, we hypothesised that in the brain stimulation condition resource depletion would decrease due to the putatively enhanced excitability of the prefrontal cortex. In other words, we expected that brain stimulation would eliminate the effect of phase I on phase II task performance. In Experiment 2, we aimed to replicate our findings from Experiment 1 in an independent second sample. We also added an additional condition (i.e., cathodal tDCS).

## Experiment 1 – Method

### Participants

Thirty-six university students (16 males; *mean age* = 20.97; min18/max26 years; *SD* = 1.91) took part in Experiment 1. The sample size was based on previous tDCS studies of similar design. They had no history of neurological or psychiatric disorders and gave their written informed consent prior to participation. Experiment 1 was approved by the local Ethical Committee of the Faculty of Psychology and Neuroscience, Maastricht University. Participants were recruited at Maastricht University through subject data bases, e-mails, Facebook, and flyers.

### Design

**Fig 1:**
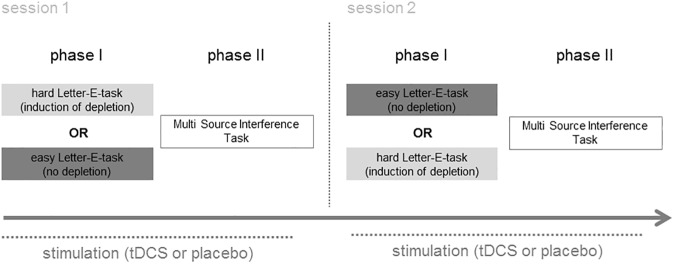
Experimental procedure. Each of the two experimental sessions consisted of phase I, in which low or high depletion was induced (by an easy versus hard Letter-E-task), and phase II, in which the remaining regulatory capacity after induction of depletion was measured evaluating reaction times (in a Multi Source Interference Task). Whether participants had to perform the depletion-inducing phase I task in session 1 or session 2 was fully randomised. The experimental group received andoal transcranial Direct Current Stimulation (tDCS); the control group received placebo stimulation (placebo and anodalin experiment 2). Stimulation was applied for 1650 seconds during the entire pahses I and II.

Participants were randomly assigned to one of two brain stimulation conditions: real tDCS enhancing the right dorsolateral prefrontal cortex (*n* = 18) or placebo tDCS (*n* = 18). During phase I of each experimental session, variable depletion was induced; participants performed either a task involving high regulatory control or a conceptually similar task involving relatively low regulatory control. All participants completed both forms of induction of regulatory resource depletion in two different experimental sessions. The order of these sessions was counterbalanced and included in the analysis as separate factor to control for a potential order effect. The experimental design was comprised of a 2 between (Brain Stimulation: real tDCS versus placebo tDCS) x 2 within (Regulatory Resource Depletion: induction of high depletion versus induction of low depletion) x 2 within (Session Order: high regulatory control then low regulatory control in phase I versus low regulatory control then high regulatory control in phase I) mixed factorial design (Fig 1). Both, the phase I and II tasks took 8–9 minutes each. The time period between the two tasks never exceeded two minutes. Including all necessary preparations, form filling, and tDCS setup one experimental session took just below one hour. Participants came back for their second session between 6–10 days after their first.

### Procedure

#### Letter-E-task

Regulatory resource depletion was investigated employing a classic dual-phase paradigm [1]. In phase I either an easy or a hard Letter-E-task was administered. English words were presented and participants had to react by button press to either every word containing an ‘e’ (easy condition–low regulatory control) or to every word containing an ‘e’, which was at least two positions away from any other vowel (hard condition–high regulatory control). This task has previously been used successfully to induce high versus little to no depletion [12].

#### Multi Source Interference Task (MSIT)

In phase II of each experimental session, participants had to perform an MSIT [12]. Three numbers were presented on a screen, of which two were identical and one was singular. Participants had to indicate the numeric value of the singular number (one–right index finger, two–right middle finger or three–right ring finger) independent of the number’s spatial position on the screen (position one–left, position two–middle or position three–right). In congruent trials the numeric amount corresponded to the spatial position of the singular number, the accompanying numbers were zeros, and the target was written in larger font than the distractor stimuli. In incongruent trials the numeric amount of the singular number did not correspond to its spatial position, the accompanying numbers were ones, twos or threes and displayed in a bigger font than the target number. Thus, three types of interference (spatial position, accompanying numbers and font size) were integrated into incongruent trials. The outcome measure of the MSIT was the mean reaction time (RT). Participants always completed training-trials before performing a task.

#### Questionnaires

Subjective fatigue, mood and arousal were assessed three times in the course of each experimental session (t1: before phase I, t2: between phase I and phase II, t3: after phase II). Fatigue was assessed using a shortened version of the fatigue questionnaire ([18]; 12 items e.g. 'how sleepy, tired, fatigued … are you'). Mood (happy to sad) and arousal (calm to nervous) were assessed with the Self-Assessment Mannequin [19].

#### TDCS

**Fig 2:**
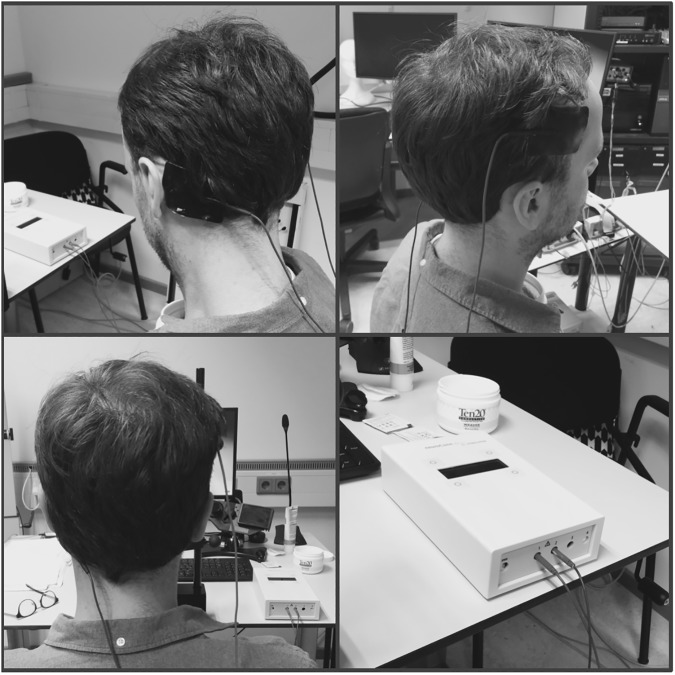
TDCS setup. Anode over F4, cathode over contralateral mastoid.

The anodal electrode was positioned over F4 (international 10-20-EEG-system), while the cathode was positioned over the contralateral mastoid (Fig 2). A DC-stimulator plus and 5x7cm electrodes (neuroConn, Ilmenau, Germany) were used. We induced 2.0 milliampere direct current during the entire experimental session (ramping up and down phases of 30 seconds each). We chose to apply stimulation throughout the entire experiment (i.e. during the entire phases I and II; 1650 seconds in total). Our rational was to modulate actual depletion, which is assumed to take place during phase I. At the same time, we wanted to avoid confounding our results due to immediate tDCS after effects, which might have occurred if stimulation was only applied during phase I, but not phase II. Finally, we wanted to control for inducing a mere general enhancement of cognitive control in phase II, instead of actually decreasing depletion. By stimulating during both phases I and II, a general enhancement of cognitive control would have occurred during both phases equally, and thus be controlled for.

In the placebo condition, the same procedure was followed, but the stimulation was switched off immediately after the ramping phases. This mimicked the skin sensation accompanying tDCS application deceiving participants about the condition they were assigned to. To ensure that conditions could not be differentiated, participants had to indicate their confidence for having either received real or placebo stimulation on a 7-point Likert Scale (from 1 ‘for sure placebo’ to 7 ‘for sure real’ with 4 ‘no idea’). During brain stimulation, participants performed both the respective phase I task (easy or hard Letter-E-task) and the MSIT in phase II.

### Data analysis

Statistical analyses were performed using SPSS19 (IBM Statistics, USA). Of originally thirty-nine participants, three were excluded, because of outlier analysis regarding the outcome variable (mean reaction times MSIT; analyses performed according to the outlier labeling rule; [20]). One additional participant was excluded, because he/she committed twice the number of mistakes compared to the worst performing participant in the MSIT task and experimenters were concerned about his/her basic understanding of the task. Thirty-five participants could be included in the analysis (tDCS *n* = 18, placebo stimulation *n* = 17). Pairs of means were compared using independent or paired-sample t-Tests. Correlations were investigated by calculating bivariate Pearson correlations. To test the effect of brain stimulation on regulatory resource depletion, an analysis of variance (ANOVA) was conducted including one or two between-subject factor (Brain Stimulation: anodal tDCS versus placebo stimulation, Session Order: high regulatory control then low regulatory control in phase I versus low regulatory control then high regulatory control in phase I) and one within-subject factor (Regulatory Resource Depletion: high regulatory control in phase I versus low regulatory control in phase I), accounting for the performance on the depleting task (mean RT on hard Letter-E-task) as a covariate. This covariate was included as a proxy for general inter-subject traits in skill at tasks. To test the effect of brain stimulation on subjective fatigue, two additional analyses of variance were conducted including one between-subject factor (Brain Stimulation: anodal tDCS versus placebo stimulation) and one within-subject factor (Fatigue Across Time: t1 before phase I versus t2 between phase I and t3 phase II versus after phase II).

## Experiment 1 – Results

### Manipulation check

Participants could not differentiate to which brain stimulation condition they were assigned (MEAN tDCS = 4.76, *SD* = 1.48 / MEAN placebo = 4.06, *SD* = 1.552, *t*(33) = 1.38 *p* = .176). The hard letter-E-task was significantly harder than the easy version, which was reflected by slower reaction times and increased error rates (experimental group: mean RT *t*(16) = -11.3627 *p* < .001 / mean misses *t*(16) = -3.659 *p* = .002 / mean false alarms *t*(16) = -3.071 *p* = .007; control group: mean RT *t*(17) = -9.594 *p* < .001 / mean misses *t*(17) = -4.672 *p* < .001 / mean false alarms *t*(17) = -4.262 *p* = .001)

### Self-control performance

**Fig 3:**
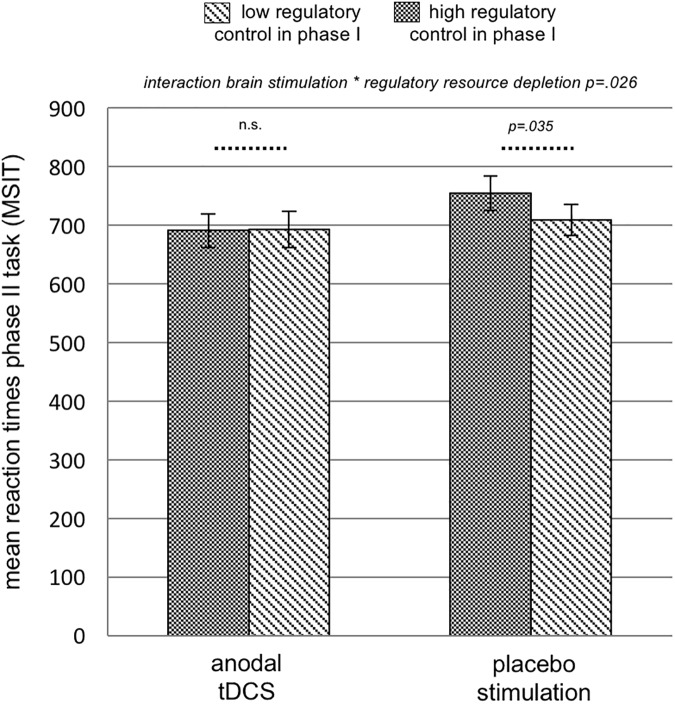
Mean reaction times on phase II task (Multi Source Interference Task) measured in ms split for groups. **Experiment 1.** We observed regulatory resource depletion in the group receiving placebo stimulation: participants were slower in phase II after having completed a previous task demanding high regulatory control versus low regulatory control. This effect was eliminated by anodal stimulation of the right dorsolateral prefrontal cortex (mixed ANOVA interaction brain stimulation * regulatory resource depletion p = .026, partial η2 = .145). n.s.: not significant (p>.05).

In the control group, we observed resource depletion, i.e. a deceleration of reaction times in phase II after performing the hard as compared to the easy Letter-E-task in phase I (*t*(17) = 2.247 *p* = .035; Table 1; Fig 3). No such effect was observed in the group receiving real tDCS (*t*(16) = -.076 *p* = .941; Table 1; Fig 3).

**Table 1:** Experiment 1. Mean reaction times (MEAN) and standard deviations (SD) of all correct trials in the phase I (Letter-E-Task) and the phase II task (MSIT). tDCS: transcranial Direct Current Stimulation; rDLPFC: right dorsolateral prefrontal cortex; MSIT: Multi Source Interference Task.

N = 35	phase I task / Letter-E-task				phase II task / MSIT			
	*anodal tDCS rDLPFC*		*placebo tDCS*		*anodal tDCS rDLPFC*		*placebo tDCS*	
	MEAN	SD	MEAN	SD	MEAN	SD	MEAN	SD
*high regulatory control*	1075.68	205.45	1178.35	254.15	691.22	28.71	754.46	29.02
*low regulatory control*	594.18	87.23	601.12	74.79	692.75	30.40	709.35	26.19

### Effects of brain stimulation on regulatory resource depletion

A 2 between (Brain Stimulation) x 2 within (Regulatory Resource Depletion) mixed ANOVA with performance (mean reaction times) on the hard Letter-E-task as covariate, revealed a main effect of Regulatory Resource Depletion (*F*(1,33) = 8.722 *p* = .006, partial *η*^*2*^ = .214; deceleration after induction of depletion) and the predicted interaction of Regulatory Resource Depletion * Brain Simulation (*F*(1,33) = 5.441 *p* = .026, partial *η*^*2*^ = .145; no deceleration after induction of depletion during tDCS). To check for order effects, the order of sessions (Session Order: high regulatory control then low regulatory control in phase I versus low regulatory control then high regulatory control in phase I) was included as an additional factor in the analysis. This revealed an interaction of Regulatory Resource Depletion * Session Order (*F*(1,33) = 14.328 *p* = .001, partial *η*^*2*^ = .323), but still an interaction of induction of regulatory resource depletion * brain stimulation condition (*F*(1,33) = 4.287 *p* = .047, partial *η*^*2*^ = .125).

### Effects of brain stimulation on phase I task

**Fig 4:**
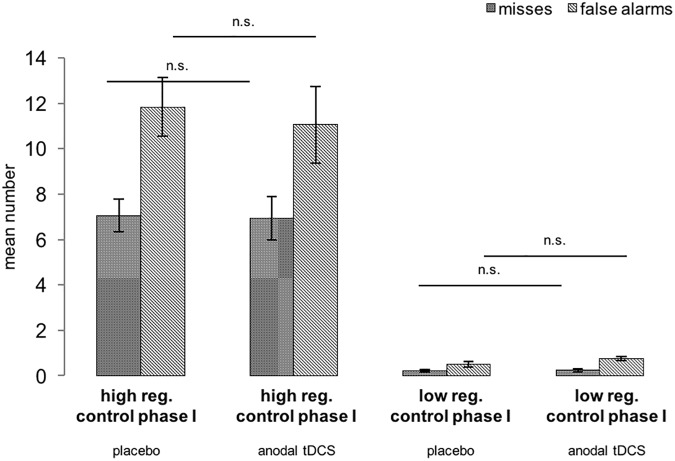
Mean number of misses, false alarms and standard deviations (SD) for phase I (Letter-E-Task). **Experiment 1.** n.s.: not significant (p>.05).

Brain stimulation did not affect the performance in phase I (Fig 4). No differences in reaction times regarding the Letter-E-task were detected in either the hard (*t*(33) = -1.309 *p* = .199) or the easy (*t*(33) = -.253 *p* = .802) version. Furthermore, participants committed the same amount of misses (hard: *t*(33) = -.048 *p* = .962 / easy: *t*(33) = .064 *p* = .950) and false alarms (hard: *t*(33) = -.183 *p* = .856 / easy: *t*(33) = .856 *p* = .398) independent of which brain stimulation condition they were assigned to.

### Control variables

Mood, arousal, and fatigue after the phase I task did not differ with respect to the induction of high versus low depletion (Table 2; mood: *t*(34) = -.421 *p* = .676; arousal: *t*(34) = 1.234 *p* = .226; fatigue: *t*(34) = 1.644 *p* = .109). None of the three control variables correlated with the size of the depletion effect (difference between MSTI mean RT in high regulatory control condition minus MSTI mean RT in low regulatory control condition; mood: *r* = .292 *p* = .088; arousal: *r* = -.197 *p* = .256; fatigue: *r* = .105 *p* = .549).

**Table 2:** Experiment 1. Means (MEAN) and standard deviations (SD) of subjective ratings regarding mood, arousal and fatigue at three time points (t1, t2, t3) during each experimental session. All variables assessed on a 7-point-Likert-scale (1 ‘happy’ vs. 7 ‘unhappy’, 1 ‘calm’ vs. 7 ‘nervous’, 1 ‘not at all tired’ vs. 7 ‘extremely tired’). tDCS: transcranial Direct Current Stimulation; rDLPFC: right dorsolateral prefrontal cortex; reg.: regulatory.

		before phase I / t1						between phase I & phase II / t2						after phase II / t3					
		*mood*		*arousal*		*fatigue*		*mood*		*arousal*		*fatigue*		*mood*		*arousal*		*fatigue*	
		*MEAN*	*SD*	*MEAN*	*SD*	*MEAN*	*SD*	*MEAN*	*SD*	*MEAN*	*SD*	*MEAN*	*SD*	*MEAN*	*SD*	*MEAN*	*SD*	*MEAN*	*SD*
***placebo tDCS***	*high reg*. *control*	**5.78**	**.81**	**2.00**	**.69**	**2.37**	**.71**	**5.67**	**.84**	**1.89**	**.83**	**2.90**	**1.00**	**5.89**	**.68**	**1.72**	**.67**	**3.41**	**1.38**
	*low reg*. *control*	**5.83**	**.71**	**1.83**	**1.04**	**2.36**	**.68**	**5.89**	**.83**	**1.67**	**.91**	**2.54**	**.89**	**5.89**	**.83**	**1.56**	**.86**	**3.07**	**1.20**
***anodal tDCS rDLPFC***	*high reg*. *control*	**5.71**	**.85**	**2.06**	**.66**	**2.23**	**.52**	**5.53**	**.80**	**1.88**	**.78**	**2.26**	**.73**	**5.59**	**.87**	**1.71**	**.59**	**2.51**	**.96**
	*low reg*. *control*	**5.71**	**.67**	**1.88**	**.78**	**2.12**	**.53**	**5.41**	**.87**	**1.76**	**.75**	**2.24**	**.55**	**5.53**	**1.07**	**1.76**	**.75**	**2.25**	**.67**

### Effects of brain stimulation on fatigue

**Fig 5:**
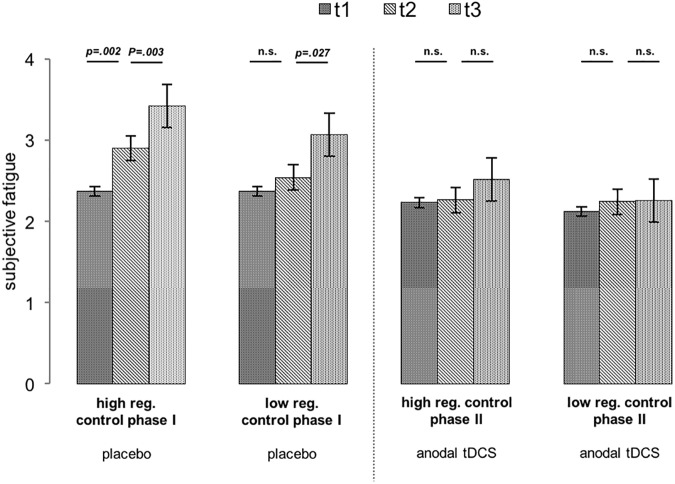
Increase of subjective fatigue over time. **Experiment 1.** Participants’ subjective fatigue levels increased over the course of each experimental session when receiving placebo stimulation. This increase in subjective fatigue was prevented by anodal stimulation to the right dorsolateral prefrontal cortex (mixed ANOVA interaction brain stimulation * subjective fatigue high regulatory control: p = .007, partial η2 = .14; low regulatory control: p = .013, partial η2 = .124). t1: before phase I, t2: between phase I and phase II, t3: after phase II. n.s.: not significant (p>.05).

Fatigue was not related to regulatory resource depletion, but it was affected by brain stimulation (Fig 5). A 3 within (Fatigue Across Time: t1 before phase I versus t2 between phase I and t3 phase II versus after phase II) x 2 between (Brain Stimulation: anodal tDCS versus placebo stimulation) mixed ANOVA revealed a main effect of fatigue (high regulatory control in phase I: *F*(1,33) = 16.220 *p* < .001, partial *η*^*2*^ = .330 / low regulatory control in phase I: *F*(1,33) = 8.196 *p* = .001, partial *η*^*2*^ = .199) and an interaction Fatigue Across Time * Brain Stimulation (high regulatory control in phase I: *F*(1,33) = 5.429 *p* = .007, partial *η*^*2*^ = .141 / low regulatory control in phase I: *F*(1,33) = 4.664 *p* = .013, partial *η*^*2*^ = .124; less fatigue during tDCS).

Summarizing Experiment 1, using a classical dual-phase paradigm we found that performance on a task demanding high self-regulation in phase 2 was significantly impaired when being preceded by a high as compared to a low self-regulation demanding task in phase 1. In our data set, this depletion effect was not related to changes in mood, arousal, or fatigue. In accordance with our hypotheses, enhancing neural excitability levels within the right dorsolateral prefrontal cortex by means of anodal tDCS significantly reduced the observed regulatory resource depletion, while leaving performance in the depleting task unaffected.

### Experiment 2

One methodological limitation of Experiment 1 lies in the control condition of the applied noninvasive brain stimulation. Although we demonstrate a significant modulation of the depletion effect after anodal stimulation to the right dorsolateral prefrontal cortex, we compare this active stimulation condition only with placebo stimulation. While we argue that this does not represent a methodological problem per se because in tDCS, placebo stimulation can indeed not be distinguished from real stimulation and, thus, be regarded as an appropriate control condition, it would have been inarguably even stronger to show that cathodal tDCS (i.e., stimulation of opposite polarity) to the right dorsolateral prefrontal cortex, as compared to placebo and as compared to anodal tDCS, leads to an increase in self-regulatory resource depletion.

To replicate and further strengthen our findings from Experiment 1, we repeated the procedure in a second independent sample introducing a second, complementary condition. In addition to modulating excitability in the right dorsolateral prefrontal cortex using anodal tDCS and applying placebo stimulation as a control, we also included cathodal tDCS.

## Experiment 2 – Method

### Participants

Forty-five university students (18 males; *mean age* = 22.78; min18/max35 years; *SD* = 2.95), who had not participated in Experiment 1, participated in Experiment 2. The sample size was based on previous tDCS studies of similar design. After ensuring that they had no neurological or psychiatric disorders, they signed a written informed consent form before taking part in the experiment. The local Ethical Committee of the Faculty of Psychology and Neuroscience, Maastricht University, approved Experiment 2. Participants were recruited at Maastricht University through subject data bases, e-mails, Facebook, and flyers.

### Design

Participants were randomly assigned to one of three brain stimulation conditions: anodal tDCS aiming at enhancing activity in the right dorsolateral prefrontal cortex (*n* = 16), cathodal tDCS aiming at decreasing activity in the dorsolateral prefrontal cortex (*n* = 15), or placebo tDCS (*n* = 14). The experimental procedure coincided with Experiment 1 (see above).

### Data analysis

We performed statistical analyses via SPSS19 (IBM Statistics, USA). Two participants had to be excluded as they repeatedly reported difficulties with understanding the task instructions during both experimental sessions (both participants would also have been excluded based on outlier analysis regarding the central outcome variable–i.e., phase II mean reaction times–according to the outlier labeling rule [20]). Therefore, the final analyses were based on forty-three participants (anodal tDCS *n* = 14, cathodal tDCS *n* = 15, placebo stimulation *n* = 14).

To compare means, paired-sample t-Tests were employed. Correlations were estimated with bivariate Pearson correlations. To test the effect of brain stimulation on regulatory resource depletion, an analysis of variance (ANOVA) was conducted including one between-subject factor (Brain Stimulation: anodal tDCS versus cathodal tDCS versus placebo stimulation) and one within-subject factor (Regulatory Resource Depletion: high regulatory control in phase I versus low regulatory control in phase I), accounting for the performance on the depleting phase I task (mean RT on hard Letter-E-task) as a covariate. To test the effect of brain stimulation on subjective fatigue, two additional analyses of variance were conducted including one between-subject factor (Brain Stimulation: anodal tDCS versus cathodal tDCS versus placebo stimulation) and one within-subject factor (Fatigue Across Time: t1 before phase I versus t2 between phase I and t3 phase II versus after phase II).

## Experiment 2 – Results

### Manipulation check

In the groups receiving anodal or placebo stimulation, the hard letter-E-task was significantly harder than the easy version, which was reflected by slower reaction times and increased error rates (experimental group anodal: mean RT *t*(13) = -11.037 *p* < .001 / mean misses *t*(13) = -5.126 *p* < .001 / mean false alarms *t*(13) = -3.226 *p* = .007; control group: mean RT *t*(13) = -10.150 *p* < .001 / mean misses *t*(13) = -5.147 *p* < .001 / mean false alarms *t*(13) = -2.853 *p* = .014). In the group receiving cathodal stimulation a similar tendency was observed (experimental group cathodal: mean RT *t*(14) = -9.836 *p* < .001 / mean misses *t*(14) = -2.495 *p* = .026 / mean false alarms *t*(14) = -2.823 *p* = .0140).

### Self-control performance

**Fig 6:**
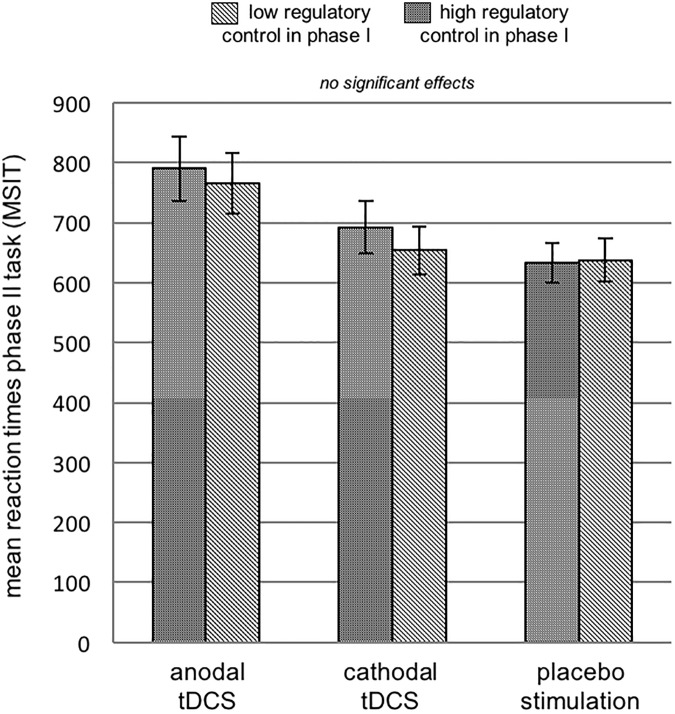
Mean reaction times on phase II task (Multi Source Interference Task) measured in ms split for groups. **Experiment 2.** Neither the basic effect of regulatory resource depletion nor any brain stimulation effect on performance in phase II could be replicated.

In the control group receiving placebo stimulation, we could not replicate resource depletion, as we did not find a deceleration of reaction times in phase II after performing the hard as compared to the easy Letter-E-task in phase I (*t*(13) = -.212 *p* = .835; Table 3; Fig 6). Neither was such an effect observed in the groups receiving anodal (*t*(13) = .934 *p* = .368; Table 1; Fig 6) nor cathodal tDCS (*t*(14) = 1.148 *p* = .270; Table 3; Fig 6).

**Table 3:** Experiment 2. Mean reaction times (MEAN) and standard deviations (SD) of all correct trials in the phase I (Letter-E-Task) and the phase II task (MSIT). tDCS: transcranial Direct Current Stimulation; rDLPFC: right dorsolateral prefrontal cortex; MSIT: Multi Source Interference Task.

N = 43	phase I task / Letter-E-task						phase II task / MSIT					
	*anodal tDCS rDLPFC*		*cathodal tDCS rDLPFC*		*placebo tDCS*		*anodal tDCS rDLPFC*		*cathodal tDCS rDLPFC*		*placebo tDCS*	
	MEAN	SD	MEAN	SD	MEAN	SD	MEAN	SD	MEAN	SD	MEAN	SD
*high regulatory control*	1130.62	190.61	1120.40	192.04	1021.48	179.85	789.99	198.76	692.13	171.13	632.93	122.51
*low regulatory control*	614.60	76.24	580.14	70.11	538.00	44.82	765.12	187.26	653.51	155.76	637.01	134.00

### Effects of brain stimulation on performance in phase II

A 3 between (Brain Stimulation) x 2 within (Regulatory Resource Depletion) mixed ANOVA with performance (mean reaction times) on the hard Letter-E-task as covariate, revealed no main effect of Regulatory Resource Depletion (*F*(1,39) = .748 *p* = .392, partial *η*^*2*^ = .019) and no interaction of phase II performance * Brain Simulation (*F*(2,39) = .354 *p* = .704, partial *η*^*2*^ = .018).

### Effects of brain stimulation on phase I task

Brain stimulation barely affected the performance in phase I (Table 3). No differences in reaction times regarding the Letter-E-task were detected in the hard (*F*(2,40) = 1.456 *p* = .245) version. In the easy version participants receiving anodal stimulation reacted significantly faster than those receiving cathodal or placebo stimulation (*F*(2,40) = 4.836 *p* = .013). Furthermore, participants committed the same amount of misses (hard: *F*(2,40) = 1.758 *p* = .186 / easy: *F*(2,40) = .616 *p* = .545) and false alarms (hard: *F*(2,40) = .339 *p* = .714 / easy: *F*(2,40) = 2.625 *p* = .085) independent of which brain stimulation condition they were assigned to.

#### Control variables

Mood, arousal, and fatigue after the phase I task did not differ with respect to whether the easy or the hard Letter-E-task was performed (Table 4; mood: *t*(42) = -.172 *p* = .864; arousal: *t*(42) = .374 *p* = .186 *p* = .710; fatigue: *t*(42) = -.154 *p* = .879).

**Table 4:** Experiment 2. Means (MEAN) and standard deviations (SD) of subjective ratings regarding mood, arousal and fatigue at three time points (t1, t2, t3) during each experimental session. All variables assessed on a 7-point-Likert-scale (1 ‘happy’ vs. 7 ‘unhappy’, 1 ‘calm’ vs. 7 ‘nervous’, 1 ‘not at all tired’ vs. 7 ‘extremely tired’). tDCS: transcranial Direct Current Stimulation; rDLPFC: right dorsolateral prefrontal cortex; reg. regulatory.

		before phase I / t1						between phase I & phase II / t2						after phase II /t3					
		*mood*		*arousal*		*fatigue*		*mood*		*arousal*		*fatigue*		*mood*		*arousal*		*fatigue*	
		*MEAN*	*SD*	*MEAN*	*SD*	*MEAN*	*SD*	*MEAN*	*SD*	*MEAN*	*SD*	*MEAN*	*SD*	*MEAN*	*SD*	*MEAN*	*SD*	*MEAN*	*SD*
***placebo tDCS***	*high reg*. *control*	**5.64**	**.74**	**2.14**	**.95**	**2.46**	**.56**	**5.71**	**.73**	**1.86**	**.77**	**7.78**	**.72**	**5.79**	**.70**	**1.79**	**.80**	**2.85**	**.89**
	*low reg*. *control*	**5.50**	**.76**	**1.93**	**.73**	**2.79**	**.84**	**5.43**	**.85**	**1.57**	**.51**	**2.86**	**.92**	**5.43**	**.85**	**1.64**	**.63**	**3.27**	**1.11**
***anodal tDCS rDLPFC***	*high reg*. *control*	**5.64**	**.93**	**2.14**	**1.23**	**2.14**	**.74**	**5.29**	**.83**	**2.00**	**.88**	**2.53**	**1.03**	**5.57**	**1.02**	**1.79**	**.58**	**3.00**	**1.18**
	*low reg*. *control*	**5.71**	**.91**	**2.64**	**1.28**	**2.10**	**.60**	**5.64**	**.74**	**2.07**	**1.07**	**2.65**	**.94**	**5.57**	**1.02**	**2.00**	**1.11**	**2.89**	**1.26**
***cathodal tDCS rDLPFC***	*high reg*. *control*	**5.53**	**1.06**	**2.53**	**.92**	**2.72**	**1.11**	**5.40**	**.91**	**2.00**	**.76**	**2.98**	**1.33**	**5.40**	**.91**	**1.80**	**.94**	**3.44**	**1.51**
	*low reg*. *control*	**5.53**	**1.06**	**2.93**	**1.58**	**2.24**	**.82**	**5.40**	**.91**	**2.06**	**.96**	**2.85**	**1.23**	**5.33**	**.98**	**2.07**	**1.10**	**3.38**	**1.31**

### Effects of brain stimulation on fatigue

As revealed by a 3 within (Fatigue Across Time) x 3 between (Brain Stimulation) mixed ANOVA, subjective fatigue ratings (Table 4) increased in the course of each session (main effect; high regulatory control in phase I: *F*(1,40) = 20.395 *p* < .001, partial *η*^*2*^ = .338 / low regulatory control in phase I: *F*(1,40) = 27.540 *p* < .001, partial *η*^*2*^ = .406), but were not associated with either depletion or brain stimulation condition (interaction; high regulatory control in phase I: *F*(2,40) = .885 *p* = .423, partial *η*^*2*^ = .042 / low regulatory control in phase I: *F*(2,40) = 1.582 *p* = .218, partial *η*^*2*^ = .073).

## Discussion

This study set out to elucidate the neural mechanisms underlying regulatory resource depletion. We hypothesised that after an act of self-regulation, performance on a subsequent self-control act will be impaired due to temporarily depleted resources (*ego depletion effect*). In addition, we hypothesised that such resource depletion arises from overstraining the prefrontal cortex. Therefore, modulating the excitability of the right dorsolateral prefrontal cortex by means of non-invasive brain stimulation should alter the depletion effect by affecting the cognitive control system.

### Experiment 1

Experiment 1 confirmed both of these hypotheses by documenting the hypothesised regulatory resource depletion effect and its significant reduction following anodal tDCS to the right prefrontal cortex.

Our classical dual-phase paradigm revealed that performance on the high self-regulation demanding task in phase 2 was significantly impaired when preceded by a high rather than a low self-regulation demanding task in phase 1. This demand- and phase-specific behavioural impairment is in line with the strength model of regulatory control [1,8]. We further showed that this ego depletion effect was not related to changes in mood, arousal, or fatigue.

As statistically confirmed by a mixed ANOVA interaction effect, enhancing excitability within the right dorsolateral prefrontal cortex reduced regulatory resource depletion, while performance in the depleting phase I task was unaffected. This suggested that the alterations in resource depletion should stem from the putative neural enhancement and not from alternated performance in the phase I task. This finding empirically supports the neural model of self-control failure by Heatherton and Wagner [11], which states that the prefrontal control system is overstrained in the case of resource depletion. Furthermore, it is in line with functional imaging [14,15] and psychopharmacological studies [12] indirectly linking prefrontal cortex and regulatory resource depletion. Our result cannot be attributed to expectancy effects, as participants could not reliably indicate whether they received tDCS or placebo stimulation. Our brain stimulation intervention in Experiment 1 modulated regulatory capacities when resources were low. When participants still had resources left at their disposal, and were thus not depleted, anodal stimulation of the prefrontal cortex did not decrease depletion. This ceiling effect might mirror on a neural level, what other studies have found with behavioural interventions [21–27]. The observed effects could not be attributed to differences in mood, arousal, and fatigue. This is in accordance with previous literature, which found resource depletion not to be related to fatigue [27] and could reflect that participants either are not adequately capable of reporting their subjective fatigue level or that resource depletion is indeed independent of an increase in subjective fatigue as suggested by the limited strength model [8].

Anodal tDCS to the right dorsolateral prefrontal cortex, furthermore, seemingly prevented the increase of subjective fatigue over time. In both conditions (high versus low regulatory demand during phase I) participants’ fatigue levels increased over the course of each experimental session when receiving placebo stimulation. This increase in fatigue was not observed during brain stimulation. This suggests that anodal stimulation of right prefrontal cortex may have affected subjective self-reports of fatigue.

Based on these results and our hypothesis-driven conclusive analyses regarding the functional role of prefrontal cortex in regulatory control and its possible resource depletion, as well as providing the first documentation of the potential for noninvasive brain stimulation to avoid or delay such ego depletion effects, it would have been standard scientific practice to aim at publishing the results of Experiment 1. The perspective to have identified a possible brain-system-based intervention to avoid ego depletion and strengthen cognitive control opens the door for wide ranging implications within and beyond our field.

### Experiment 2

To overcome the methodological limitation of a missing control condition in Experiment 1 (for outline see Method), we decided to conduct a second experiment, repeating the procedure in a second independent sample while introducing an additional condition of cathodal tDCS.

Unexpectedly in Experiment 2, we failed to replicate the basic regulatory depletion effect with our dual-phase paradigm. In other words, contrary to Experiment 1, the sample of Experiment 2 did not show a significant performance impairment in the high self-regulation demanding task of phase II when being preceded by a high as compared to a low self-regulation demanding task in phase I. Additionally, as the expected psychological depletion effect was not replicated, no significant modulatory influence of either anodal or cathodal non-invasive brain stimulation could be found.

The discrepant results in Experiment 1 and 2 could stem from insufficient power and / or differences in experimental protocol. Despite the fact that both studies are obviously based on different population samples and data acquisition partly done by different research staff, both experiments were conducted in the same laboratory under identical experimental conditions, during the same months of two consecutive years, with participants recruited from the same participant pool within one academic environment. If regulatory resource depletion according to the strength model of self-control [8] is as stable an effect as past literature suggested [1], these minor differences between both studies should, in our view, not have altered the results so dramatically.

It should be noted that reaction times differed between experiments 1 and 2, for both phase I and II tasks (see Tables 1 & 3). While participants tended to react slightly slower in both tasks during anodal stimulation in experiment II as compared to experiment I, they tended to react slightly faster during placebo stimulation. One can speculate, in particular with regard to the speeding up during placebo stimulation, on whether this might have altered the cognitive load of the tasks and thus, confounded results. As the described differences in reaction times occurred for both phases and in both conditions (high versus low regulatory control) equally and independent of brain stimulation (i.e. mostly during placebo stimulation), they cannot explain the failure of replication. They underline, however, the heterogeneity of a cognitive process which was long thought to be stable.

### (Non-)replicability of resource depletion

Interestingly, our contradictory results reflect the current scientific discussion on ego depletion [2,10]: As it is not clear, how many studies with zero findings have been conducted on regulatory resource depletion that were never published, it still remains possible–and the great similarity between the current two studies supports this option–that resource depletion is nothing more than an incidental finding caused by insufficient power. Publication bias was repeatedly identified as the main reason for an over-estimation of empirical evidence in favor of the ego depletion effect [10]. The historical tendency for negative results to be overlooked and unpublished (perhaps not even publishable [25]) motivated us to present our results. We want to be forthright in neutrally communicating our empirical procedure which was designed in good faith and based on sound methodology.

Given the mixed results across our studies, we cannot firmly argue for or against the existence of regulatory resource depletion. Instead, we aim to identify what questions might explain the inconsistent findings that we and other groups have recently reported. Certainly, we cannot assume that regulatory resource depletion as described by the strength model of self-control is a fundamental psychological effect. If it does exist, it is likely of unstable nature and susceptible to a wide range of external and internal factors, such as self-monitoring [21,26], mood [27] and motivation [24]. The fact that the two current experiments were very similar, raises the possibility that resource depletion is affected by individual or environmental differences that have not been accounted for. Possibly, there are one or multiple personality traits that cause an individual to show stronger depletion effects. Previous studies showing the effect may have suffered from low power and unsuccessful randomization, which in turn may cause people that are more prone to ego-depletion effects to be overrepresented. If so, then one option would be to drastically increase the sample size of studies testing the phenomenon. It has to be emphasized that an effect which cannot be replicated across independent samples, might be replicable within one sample, especially if trait variables are crutial moderators. In any case, the field would profit from investigating under which specific circumstances (if any) regulatory resource depletion occurs, along with individual differences in the degree to which the effect is expressed. Answering these questions may i) offer some explanation for variability in findings across studies, and ii) advance our theoretical models on self-control. Following suggestions by Sripada and collegues [28], we advocate a framework of analysis and scientific reasoning which extend beyond mere dichotomous outcomes of ‘replication’ versus ‘failure to replicate’. On the one hand, the manyfold differences between studies, which are also relevant to the 23 samples which were included in the recent replication study [2], should not be neglected, but taken as pieces of valuable information to further advance theoretical frameworks. On the other hand, advanced statistical modelling, including instruments such as Bayesian Factor Tests for Replication Success [29], are valuable to further identify potential underpinnings of partially replicable effects.

### (Non-)replicability of tDCS effects

Contradicting our results in Experiment 1, we did not find any modulatory effect of tDCS on perfornmance in Experiment 2. In light of the fact that the basic psychological effect on which our hypothesis was based (i.e. resource depletion) was not replicated in experiment 2, we cannot judge the extent to which the tDCS effect we originally found, is replicable. Based on the presented evidence, we demonstrated that anodal tDCS over rDLPFC decreased resource depletion and subjective fatigue, when the phenomenon actually occured. When it did not occur, no tDCS effect on any variables measuring performance could be observed. This leads to the assumption that stimulation of the right prefrontal cortex might be a promising tool to be further investigated in the context of self-control enhancement, once the specific circumstances triggering depletion are understood. These results must continue to be interpreted with caution, while considering accumulating evidence which suggests that the actual mode of action tDCS has on neuronal level is far from understood. Multiple parameters, including montage, electrode size, tissue properties, etc., might challenge the oversimplified assumption that anodal stimulation enhances, while cathodal stimulation decreases, cortical excitability (AeCi; [30]).

### The file drawer effect

The inconsistency of our findings, measured by the *p* < .05 metric, raises questions about the general trustworthiness of established scientific knowledge that is based on the publication of single studies. In current practice, there is a strong risk of “file-drawer” effects, where investigators conduct an experiment once and publish the results if they confirm a prior hypothesis, and if not, move on to the next project. A potential solution to this challenge is for researchers to run internal replications prior to publication. The additional investment of time and effort in running these replications is discouraged by the scientific reward system, as this approach often leads to inconsistent findings that are difficult to publish. Not focusing on replicability, though, leads to a dissipation of scientific resources, when future work is based on prematurely published empirical data. These incentive structures likely contribute to the ‘replication crisis’ [3] in psychology (and social psychology in particular). These concerns constantly have triggered systematic orchestrated replication projects such as the Open Science Collaboration (OSC) on estimating the reproducibility of psychological science [31]. The results from this project suggest that merely forty percent of the classic psychological phenomena are replicable, which has caused wide-spread discussion [32]. Experiencing replication challenges first hand within our laboratory has taught us, i) that it is not only worthwhile but crucial to invest in replicating one’s own work, ii) that researchers would profit scientifically, statistically (with respect to correct meta-analyses), and personally, if studies with null outcomes or ambiguous results were published more frequently and therefore, iii) that replication efforts should be strongly rewarded in the scientific community.

### Conclusion & prospective outlook

We set out to elucidate the neural mechanism underlying regulatory resource depletion in a well-controlled empirical study consisting of two experiments. While in Experiment 1, we could seemingly demonstrate a general effect of regulatory resource depletion, which was eliminated by anodal tDCS, in Experiment 2, we failed to replicate these effects in a second independent sample. Our results are a prime micro-example of the ‘replication crisis’ [3] within one coherent experimental framework. In this report, we aim to fulsomely share our ambiguous findings in the hope that it furthers discourse such that future work in the field will enable a shift in theoretical focus necessary to advance our current models of self-control. As discussed above, one option to explain our results is that the phenomenon of regulatory resource depletion simply does not exist and is, thus, not replicable. Another explanatory framework is that the effect might exist, but that its replicability is highly vulnerable to unidentified circumstantial personal and environmental variables. In this case, it is of utmost importance for the field to identify exactly what these variables are. In light of our study, this might mean that once the basic effect occurs (i.e., all not yet known factors necessary to induce resource depletion are present), the effect is modulated by excitability within the right dorsolateral prefrontal cortex. Therefore, we still argue that anodal stimulation of right prefrontal cortex by means of tDCS might be a promising tool to be further looked into in the context of self-control enhancement.

## References

[1] Hagger M. S., Wood C., Stiff C., & Chatzisarantis N. L. D. (2010). Ego depletion and the strength model of self-control: a meta-analysis. Psychological Bulletin, 136(4), 495–525. 10.1037/a0019486 20565167

[2] Hagger M. S., Chatzisarantis N. L., Alberts H., Anggono C. O., Batailler C., Birt A., et al (2015). A multi-lab pre-registered replication of the ego-depletion effect. Perspectives on Psychological Science, 2. 10.1177/1745691616652873 27474142

[3] Schooler J. W. (2014). Metascience could rescue thereplication crisis'. Nature, 515, 9 10.1038/515009a 25373639

[4] Tangney J. P., Baumeister R. F., & Boone A. L. (2004). High self‐control predicts good adjustment, less pathology, better grades, and interpersonal success. Journal of personality, 72(2), 271–324. 15016066 10.1111/j.0022-3506.2004.00263.x

[5] Baumeister R. F., Heatherton T. F., & Tice D. M. (1994). Losing control: How and why people fail at self-regulation Academic press.

[6] Muraven M., & Baumeister R. F. (2000). Self-regulation and depletion of limited resources: does self-control resemble a muscle? Psychological Bulletin, 126(2), 247–259. 10748642 10.1037/0033-2909.126.2.247

[7] Wills T. A., & Stoolmiller M. (2002). The role of self-control in early escalation of substance use: a time-varying analysis. Journal of consulting and clinical psychology, 70(4), 986 12182282 10.1037//0022-006x.70.4.986

[8] Baumeister R. F., Bratslavsky E., Muraven M., & Tice D. M. (1998). Ego depletion: is the active self a limited resource? Journal of Personality and Social Psychology, 74(5), 1252–1265. 9599441 10.1037//0022-3514.74.5.1252

[9] Schmeichel B. J., Volokhov R. N., & Demaree H. A. (2008). Working memory capacity and the self-regulation of emotional expression and experience. Journal of Personality and Social Psychology, 95(6), 1526–40. 10.1037/a0013345 19025300

[10] Carter E. C., & McCullough M. E. (2014). Publication bias and the limited strength model of self-control: has the evidence for ego depletion been overestimated?. Frontiers in psychology, 5, 823 10.3389/fpsyg.2014.00823 25126083 PMC4115664

[11] Heatherton T. F., & Wagner D. D. (2011). Cognitive neuroscience of self-regulation failure. Trends in Cognitive Sciences, 15(3), 132–9. 10.1016/j.tics.2010.12.005 21273114 PMC3062191

[12] Sripada C., Kessler D., & Jonides J. (2014). Methylphenidate Blocks Effort-Induced Depletion of Regulatory Control in Healthy Volunteers. Psychological Science, 25(6), 1227–1234. 10.1177/0956797614526415 24756766 PMC4206661

[13] Solanto M. V. (2002). Dopamine dysfunction in AD/HD: Integrating clinical and basic neuroscience research. In Behavioral Brain Research (Vol. 130, pp. 65–71). 10.1016/s0166-4328(01)00431-4 11864719

[14] Richeson J. A., Baird A. A., Gordon H. L., Heatherton T. F., Wyland C. L., Trawalter S., et al (2003). An fMRI investigation of the impact of interracial contact on executive function. Nature Neuroscience, 6(12), 1323–1328. 10.1038/nn1156 14625557

[15] Wagner D. D., & Heatherton T. F. (2013). Self-regulatory depletion increases emotional reactivity in the amygdala. Social Cognitive and Affective Neuroscience, 8, 410–417. 10.1093/scan/nss082 22842815 PMC3624961

[16] Nitsche M. A., Cohen L. G., Wassermann E. M., Priori A., Lang N., Antal A., et al (2008). Transcranial direct current stimulation: State of the art 2008. Brain Stimulation, 1(3), 206–23. 10.1016/j.brs.2008.06.004 20633386

[17] Paulus W. (2011). Transcranial electrical stimulation (tES—tDCS; tRNS, tACS) methods. Neuropsychological Rehabilitation, 21(5), 602–17. 10.1080/09602011.2011.557292 21819181

[18] Lee K. A., Hicks G., & Nino-Murcia G. (1991). Validity and reliability of a scale to assess fatigue. Psychiatry Research, 36, 291–298. 2062970 10.1016/0165-1781(91)90027-m

[19] Bradley M. M. (1994). Measuring emotion: The self-assessment manikin and the semantic differential. Journal of Behavior Therapy and Experimental Psychiatry, 25(I), 49–59. 7962581 10.1016/0005-7916(94)90063-9

[20] Hoaglin D. C., & Iglewicz B. (1987). Fine-Tuning Some Resistant Rules for Outlier Labeling. Journal of the American Statistical Association, 82(400), 1147–1149.

[21] Alberts H. J. E. M., Martijn C., & de Vries N. K. (2011). Fighting self-control failure: Overcoming ego depletion by increasing self-awareness. Journal of Experimental Social Psychology, 47(1), 58–62.

[22] Alberts H. J. E. M., Martijn C., Greb J., Merckelbach H., & de Vries N. K. (2007). Carrying on or giving in: the role of automatic processes in overcoming ego depletion. The British Journal of Social Psychology / the British Psychological Society, 46, 383–399. 10.1348/014466606X130111 17565788

[23] Muraven M., Baumeister R. F., & Tice D. M. (1999). Longitudinal improvement of self-regulation through practice: building self-control strength through repeated exercise. The Journal of Social Psychology, 139(4), 446–457. 10.1080/00224549909598404 10457761

[24] Muraven M., & Slessareva E. (2003). Mechanisms of self-control failure: motivation and limited resources. Personality and Social Psychology Bulletin, 29, 894–906. 10.1177/0146167203029007008 15018677

[25] Sterling T. D. (1959). Publication decisions and their possible effects on inferences drawn from tests of significance—or vice versa. Journal of the American statistical association, 54(285), 30–34.

[26] Wan E. W., & Sternthal B. (2008). Regulating the effects of depletion through monitoring. Personality and Social Psychology Bulletin, 34(1), 32–46. 10.1177/0146167207306756 17975255

[27] Tice D. M., Baumeister R. F., Shmueli D., & Muraven M. (2007). Restoring the self: Positive affect helps improve self-regulation following ego depletion. Journal of Experimental Social Psychology, 43(3), 379–384.

[28] Sripada C., Kessler D., & Jonides J. (2016). Sifting signal from noise with replication science. Perspectives on Psychological Science, 11(4), 576–578. 10.1177/1745691616652875 27474144

[29] Verhagen J., & Wagenmakers E. J. (2014). Bayesian tests to quantify the result of a replication attempt. Journal of Experimental Psychology: General, 143(4), 1457. 24867486 10.1037/a0036731

[30] Jacobson L., Koslowsky M., & Lavidor M. (2012). tDCS polarity effects in motor and cognitive domains: a meta-analytical review. Experimental brain research, 216(1), 1–10. 10.1007/s00221-011-2891-9 21989847

[31] Open Science Collaboration. (2015). Estimating the reproducibility of psychological science. Science, 349(6251), aac4716 10.1126/science.aac4716 26315443

[32] Anderson C. J., Bahník Š., Barnett-Cowan M., Bosco F. A., Chandler J., Chartier C. R., et al (2016). Response to comment on “estimating the reproducibility of psychological science”. Science, 351(6277), 1037–1037. 10.1126/science.aad9163 26941312

